# Monocular catadioptric panoramic depth estimation via improved end-to-end neural network model

**DOI:** 10.3389/fnbot.2023.1278986

**Published:** 2023-09-25

**Authors:** Fei Yan, Lan Liu, Xupeng Ding, Qiong Zhang, Yunqing Liu

**Affiliations:** ^1^School of Electronic Information Engineering, Changchun University of Science and Technology, Changchun, China; ^2^New Technology Research Department, Jilin Provincial Science and Technology Innovation Center of Intelligent Perception and Information Processing, Changchun, China

**Keywords:** catadioptric panoramic camera, panoramic image, depth estimation, attention model, depth smoothness loss

## Abstract

In this paper, we propose a monocular catadioptric panoramic depth estimation algorithm based on an improved end-to-end neural network model. First, we use an enhanced concentric circle approximation unfolding algorithm to unfold the panoramic images captured by the catadioptric panoramic camera and then extract the effective regions. In addition, the integration of the Non-local attention mechanism is exploited to improve image understanding. Finally, a depth smoothness loss strategy is implemented to further enhance the reliability and precision of the estimated depths. Experimental results confirm that this refined algorithm is capable of providing highly accurate estimates of object depth.

## 1. Introduction

Traditional camera systems are often limited by their narrow field of view, a problem that is currently being alleviated by the introduction of panoramic cameras (Svoboda et al., 1998). There are four main types of panoramic vision cameras: pan-tilt rotating, fisheye lens, multi-camera stitching, and catadioptric. In particular, a catadioptric panoramic camera uses a special type of mirror, called a catadioptric mirror, to direct light from different angles onto a single image sensor, thus capturing panoramic images (Jaramillo et al., 2016). Consisting mainly of a convex reflecting mirror, an imaging lens, and a photosensitive component (Baker and Nayar, 1998, 1999), the catadioptric panoramic camera avoids the complicated designs associated with optical lens structures and solves the problem of image distortion (Liu et al., 2016). Additionally, it eliminates the call for image stitching, thus affirming the real-time capture of a 360° panoramic view.

The rapid growth of visual systems research has increasingly made panoramic vision systems a critical point of interest for researchers in related fields. This technology finds its extensive applications in areas such as robotic navigation, Internet of Things (IoT), and autonomous driving (Yamazawa et al., 1995; Liu and Liang, 2013; Khurana and Armenakis, 2018). Panoramic vision systems are designed to capture a 360° view of the environment (Nichols et al., 2010). In the field of depth estimation in panoramic vision, a depth value is computed for each pixel in an image to facilitate the approximation of distances between objects in the scene and the camera itself. Two main approaches have dominated the research field of image depth estimation: supervised and unsupervised learning.

Supervised learning is performed on datasets that are comprehensively labeled with critical depth information, providing an effective method for monocular depth estimation (Eigen et al., 2014). A unique image reconstruction loss function is incorporated to assess the disparity between the generated depth map and the input image (Li et al., 2017), thereby supporting the network's learning of image depth information. In addition, data augmentation techniques are used to amplify and transform the training data, thus diversifying the network training samples and effectively increasing the network's generalization capacity (Eldesokey et al., 2020; Kusupati et al., 2020). Despite their proven ability to deliver high-quality depth estimation results, these methods are highly dependent on the considerable time and skill of the personnel responsible for the annotation process, making the potential occurrence of annotation errors or inconsistencies virtually unmanageable.

With the advancement of deep learning techniques, unsupervised end-to-end depth estimation methods have become one of the research hotspots. End-to-end neural network models can complete the entire process from input to output without the need for human intervention at intermediate steps. These models fall into two categories: the first assimilates learning through stereo matching techniques; the second exploits the displacement between successive frames to infer the depth data associated with objects in the scene (Garg et al., 2016). The use of unlabeled monocular video sequences as network inputs to train convolutional neural networks (CNNs) in an unsupervised approach has enabled depth estimation models to be independent of labeled depth information datasets (Zhou et al., 2017). This method has expanded the potential application scenarios of depth estimation models. However, a limitation of this method is the relatively lower precision of depth estimation. Consequently, various methodologies have been adopted to enhance the performance and robustness of depth estimation. These include the employment of a reconstruction image loss function to improve the consistency between left and right disparity maps (Godard et al., 2017), and the integration of three-dimensional geometric constraints to constrain unsupervised learning of depth (Mahjourian et al., 2018). By using binary depth classification during the training process, it is possible to quickly predict nearby objects (Badki et al., 2020). In addition, even with relatively coarse quantization of depth estimation, a high level of accuracy can be maintained. To tackle the prevalent issue of unsupervised scale, joint training of monocular depth estimation and stereo visual odometry is executed through the utilization of depth information derived from stereo images relative to the motion between them (Zhan et al., 2018). Unsupervised learning methods can automatically discover the depth structure within images without the need for any manual intervention. However, the complex phenomena in real outdoor scenes, such as lighting variations, occlusions, etc., pose potential challenges to image depth estimation. During image depth estimation, these factors can potentially lead to issues such as the loss of fine details in the predicted depth map and lower accuracy in the depth map, thereby preventing the acquisition of accurate depth information.

This paper presents a novel approach to depth estimation in panoramic images using a catadioptric panoramic camera. The unique design of this camera facilitates real-time monitoring of the environment in a 360° fashion and mitigates the challenges of distortion and missing patches encountered by multiple camera systems, ultimately reducing costs. The unsupervised end-to-end depth estimation method proposed herein systematically addresses the challenge of insufficiently accurate fine detail prediction often seen in existing models. With this goal in mind, our approach incorporates a Non-local attention mechanism to capture intricate contextual dependencies within images. Additionally, we introduce a depth smoothing loss to increase the accuracy and efficiency of our depth estimation algorithm.

## 2. Proposed method

### 2.1. Catadioptric panoramic camera image preprocessing

**Figure 1:**
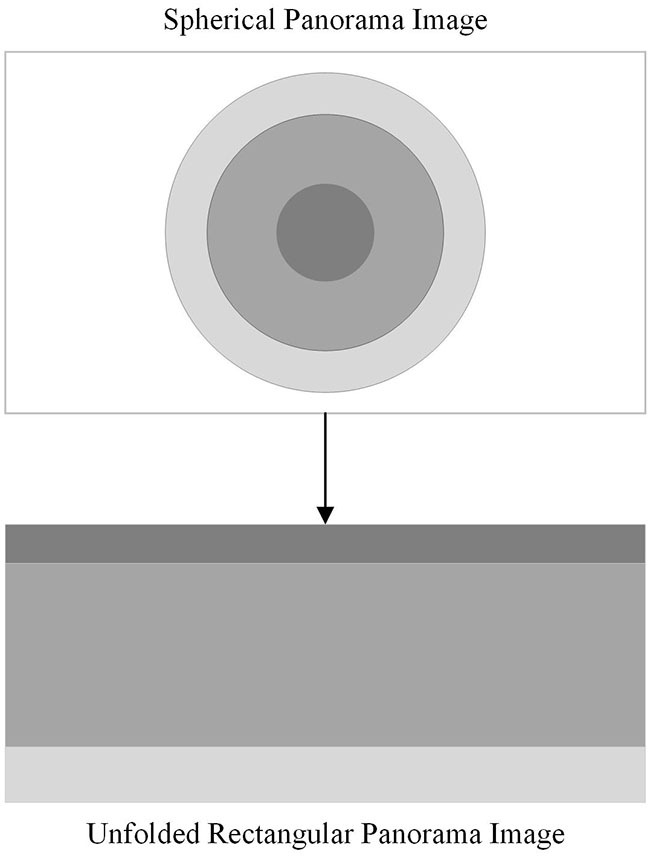
Panorama expansion.

The imaging principles, manufacturing costs, and complexity of various curved reflecting mirrors are all factors to consider when selecting reflecting mirrors for a catadioptric panoramic imaging system. A hyperbolic mirror can capture images within a broader range and it offers the advantage of lower production costs. Therefore, in this paper, hyperbolic mirrors are selected as the reflecting elements for the catadioptric panoramic imaging system. Due to the special characteristics of the imaging principle of the catadioptric panoramic camera, the panoramic image captured by the catadioptric panoramic camera has a large distortion. To solve this problem, it is necessary to expand the panoramic image into a two-dimensional rectangular image, so that each pixel in the panoramic image corresponds to a position in the expanded image. This is called panorama expansion. As shown in Figure 1.

**Figure 2:**
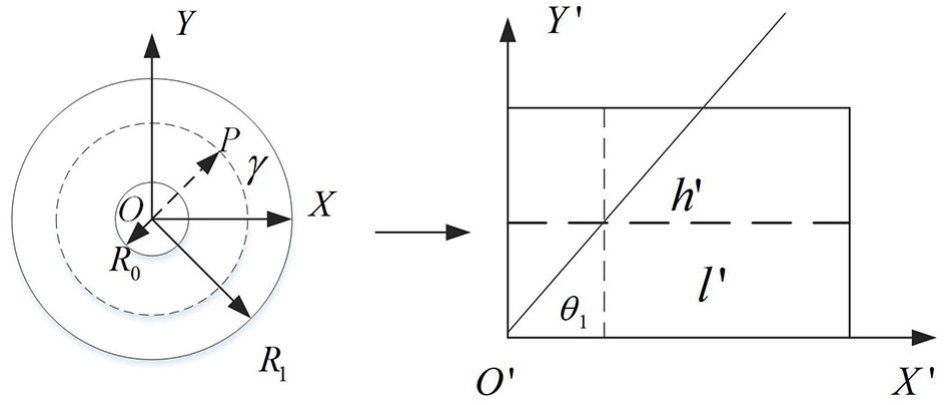
The principle of the improved concentric circle approximation unfolding algorithm.

The traditional catadioptric panorama is usually expanded by the concentric circle approximate expansion algorithm, but the distortion of the expanded image is obvious, which will affect the subsequent processing of depth estimation. Aiming at this problem, this paper improved the concentric circle approximate expansion algorithm to reduce the distortion degree of the expanded image. Figure 2 shows the principle of the improved algorithm.

A rectangular coordinate system with the center of the catadioptric panorama as the origin *O* and the horizontal and vertical directions as the *X*-axis and *Y*-axis. The dashed line in the right plot of Figure 2 is shown. Let the ring represented by the dotted line be its panorama expansion. Thus, after the panorama expansion, the length and width of the 2D rectangle can be obtained. As shown in the following formula:


(1) $$\begin{array}{l} W=l^{\prime }=2\pi r,H=h^{\prime }=r-R_{0} \end{array}$$

A ray passing through the center point *O* intersects a circular ring represented by a dashed line at a point *P*(*x*_1_, *y*_1_). After unfolding, the angle between the ray and the *X*-axis is denoted by θ_1_, so:


(2) $$\begin{array}{l} \theta_{1}=\frac{1}{R_{1}} \end{array}$$

Using ray *OP* as polar axis, rotate 360° around pole point *O*. By calculation, all the pixel values on the circumference of the circle can be obtained and arranged in a certain order. The calculation formula is:


(3) $$\left\{ \begin{array}{l} \rho =H+R_{0}\\ x=\rho \cos(\theta_{1})+u_{0}\\ y=\rho \sin(\theta_{1})+v_{0} \end{array}\right.$$

**Figure 3:**
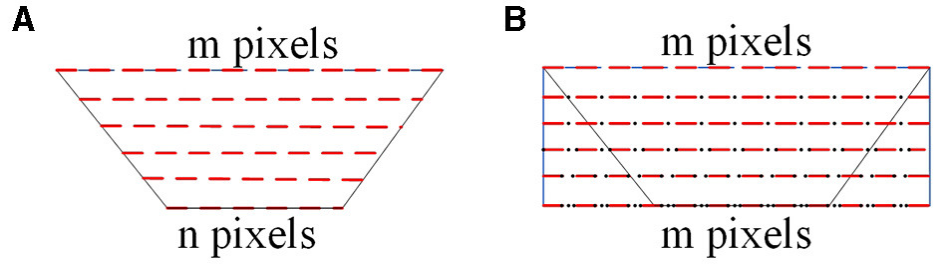
The principle of interpolation for catadioptric panoramic images. **(A)** is unfolding of the catadioptric panoramic image, and **(B)** is unfolding image after interpolation.

As shown in Figure 3, the interpolation process of the panoramic image is depicted. In Figure 3A, the red dashed lines represent the pixel values after unfolding the catadioptric panoramic image. In Figure 3B, the black dots represent the inserted pixel values. In Figure 3A, the longest side of the trapezoid corresponds to the region farthest away from the center of the catadioptric panoramic image. Based on this longest side, construct a two-dimensional rectangle, ensuring that each row has the same number of interpolated pixels as the length of the longest side. If the longest side of the trapezoidal image has m pixels, and the shortest side has n pixels, then the shortest side needs to insert m-n pixels to ensure that each row in the two-dimensional rectangle has the same number of pixels as the longest side. When performing the interpolation process, the first pixel *x*_*i*_, *i* = 1, 2, ⋯*m* of the shortest side of the trapezoidal image should be placed at the first position of the corresponding side of the rectangle. Since we need to insert *m*−*n* pixels on the shortest side, this means that between adjacent pixels, we will need to insert (*m*−*n*)/*n* pixels to maintain the required consistency in the interpolation process. By using interpolation, we insert n interpolated pixel values between adjacent pixels of the catadioptric panoramic image's shortest side. This process is performed consistently for each row, resulting in the final rectangular unfolded image of the catadioptric panoramic view. Finally, by using interpolation, we insert (*m*−*n*)/*n* pixel values between adjacent pixels of the longest side of the catadioptric panoramic image, resulting in the final rectangular unfolded image of the catadioptric panoramic view.

**Figure 4:**
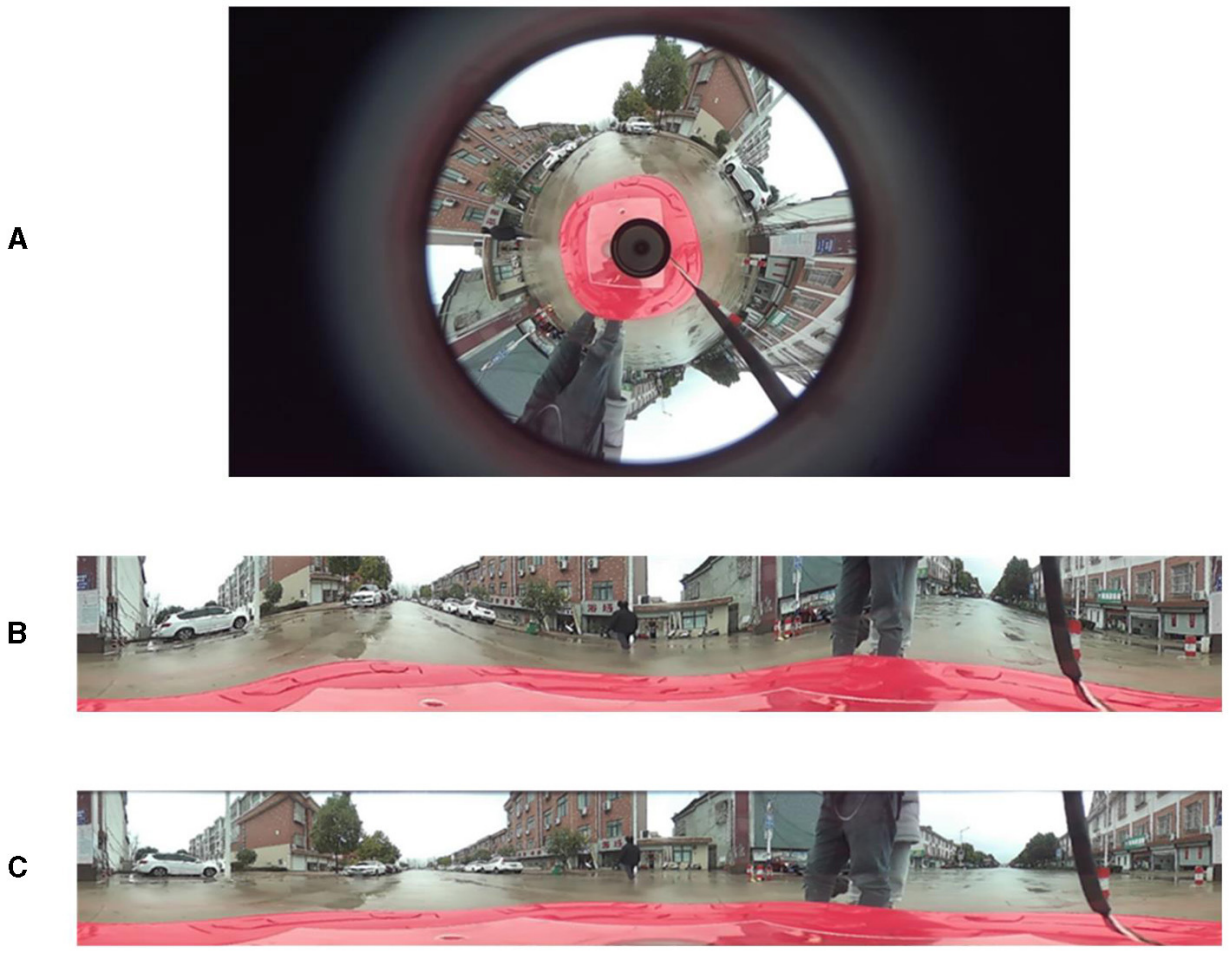
Unfolding of the catadioptric panoramic image. **(A)** is the panoramic image, **(B)** is the unfolded image using the traditional method, and **(C)** is the unfolded image using the improved method.

As shown in Figure 4, the simulation results from both methods indicate that the improved method exhibits significantly better real-time performance compared to the traditional method. Comparing the unfolded images in Figures 4B, C, the improved concentric circle approximation unfolding algorithm shows less distortion and more accurately reproduces the original scene.

**Figure 5:**
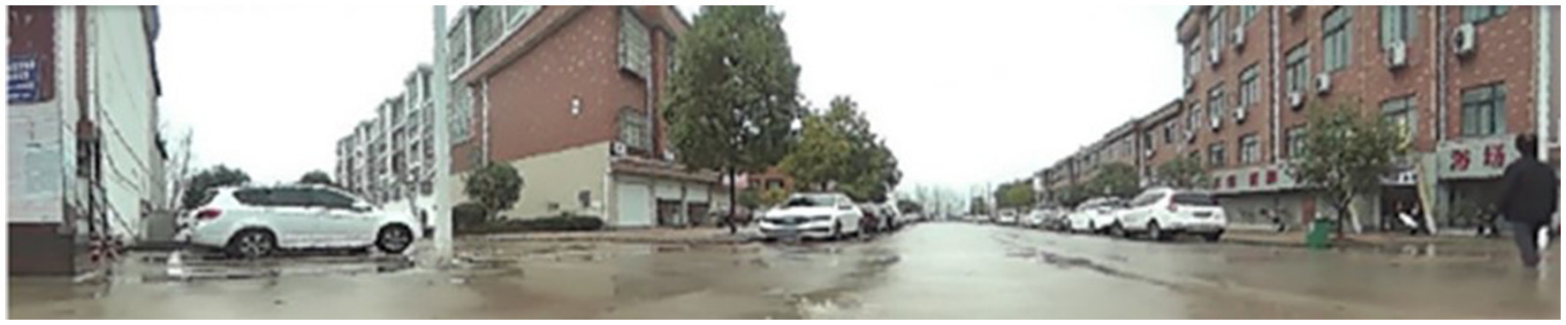
Effective region extraction.

The unfolded panoramic image contains complete 360° panoramic information of the scene. In practice, only the part of the image directly in front of the object is needed. Therefore, it is necessary to extract the relevant region from the unfolded panoramic image effectively. From the unfolded image in Figure 4C, it can be observed that the frontal view of the vehicle is located on the left side of the unfolded image, and the height of the vehicle's top part is approximately one-third of the entire image height. Therefore, the effective region of interest lies within the left-to-right half of the unfolded image and within the top-to-bottom two-thirds of the unfolded image height. As shown in Figure 5, this effective region is the crucial area for subsequent depth estimation. In fact, this can reduce the computational load, improve detection speed, and even eliminate some false positives.

### 2.2. Improved unsupervised monocular depth estimation model

To address the challenge of difficult annotation in supervised learning methods, this paper adopts an unsupervised learning approach for image depth estimation. A common problem with unsupervised end-to-end depth estimation methods is the lack of accuracy in predicting fine details. Existing unsupervised image depth estimation methods pay limited attention to the influence of the spatial context of the image on the depth information. Therefore, this paper proposes an improvement to a novel unsupervised learning algorithm framework by incorporating the Non-local attention mechanism module into the network structure of the encoder and decoder. This helps the network to perform adaptive contextual modeling for different regions in the image. This method enables the network to better comprehend various objects, backgrounds, and textures present in the image, thereby enhancing its understanding and representation capabilities of the image content.

**Figure 6:**
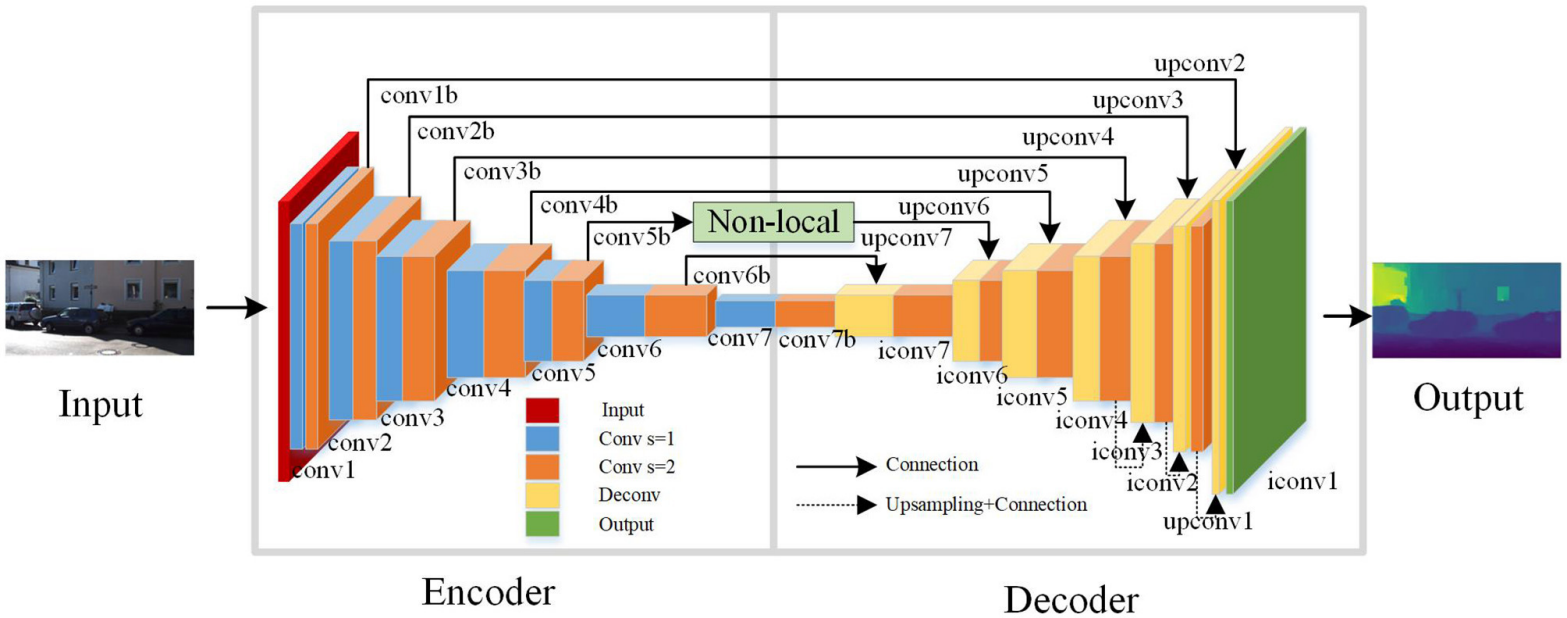
Unsupervised learning-based image depth estimation model.

As shown in Figure 6, this is the improved unsupervised learning depth estimation network model. The network is based on an end-to-end encoder-decoder framework, allowing it to perform depth estimation on images at multiple scales. In order to better capture contextual information in the image, a Non-local operation attention mechanism module is incorporated into the network framework. In each layer of the encoder, the Non-local operation attention mechanism module is used as the second operation and employs convolution with a stride of 2. The network architecture consists of three parts: an encoder, a decoder, and a Non-local operation module. The encoder is used for feature extraction, responsible for converting the input image into high-dimensional feature vectors. Convolutional neural networks (CNNs) are commonly employed to implement the encoder part. The decoder is used for depth estimation, and its main role is to decode the feature vectors extracted by the encoder into a depth map. The Non-local operation module is used to extract contextual information from the image and enables global interaction in the spatial dimension of the input feature map. This allows for the fusion of global contextual information, helping the model to better understand the relationships between objects in the image, leading to improved performance.

In this paper, the Disp Net framework (Mayer et al., 2016) is used to design the structure of the encoder. And, by combining the long-range skip connections and the Non-local operation, the network's expressive power is enhanced to obtain more accurate depth maps. Convolutional layers are mainly used for feature extraction in neural networks. Activation layers introduce nonlinearity into the neural network, which is essential for the network to learn complex and nonlinear patterns in the data. Pooling layers play a crucial role in reducing the spatial dimensions of the feature maps, which can help in reducing the computational load and the number of parameters in the network. In this network architecture, except for the output layer, ReLU activation functions are used after all the convolutional layers. That's because this activation function has advantages such as fast computation, ease of optimization, and avoidance of the vanishing gradient problem. This design strategy helps to enhance the performance and stability of the network, making the depth estimation model more reliable and practical.

In the encoder, using convolutional operations with a stride of 2 is intended to extract features more efficiently. This convolutional operation helps to reduce the size of feature maps, increase the receptive field, and decrease the number of channels, thereby reducing computational complexity, lowering memory consumption, and improving the computational efficiency of the network. Increasing the receptive field helps the network to better understand the contextual information in the input data, thereby improving the prediction accuracy of the network. Reducing the number of channels in the feature maps helps to lower the dimensionality of the data, leading to reduced computational and storage costs. By combining these operations in the encoder, the performance and efficiency of the neural network can be effectively optimized. Finally, the predicted depth values are constrained using the function 1/(α*sigmoid(*x*)+β), where α = 8 and β = 0.1. As shown in Table 1, the encoder network model's specific structure is part of an end-to-end encoder-decoder architecture.

**Table 1:** The specific structure of the encoder network model.

**Name**	**Input**	**Kernel size**	**Stride**
conv1	image	7 × 7	1
conv1b	conv1	7 × 7	2
conv2	conv1b	5 × 5	1
conv2b	conv2	5 × 5	2
conv3	conv2b	3 × 3	1
conv3b	conv3	3 × 3	2
conv4	conv3b	3 × 3	1
conv4b	conv4	3 × 3	2
conv5	conv4b	3 × 3	1
conv5b	conv5	3 × 3	2
conv6	conv5b	3 × 3	1
conv6b	conv6	3 × 3	2
conv7	conv6b	3 × 3	1
conv7b	conv7	3 × 3	2

As shown in Table 1, the decoder of the end-to-end network in this paper utilizes deconvolutional operations, taking the output of the second operation in the last layer of the encoder as its input. In the other layers of the decoder, a fusion concatenation operation is employed with the output of the second operation in the second-to-last layer of the encoder. This fusion allows the decoder to access and incorporate more image features from the encoder. The fusion concatenation operation in the other layers of the decoder follows a similar principle. Specifically, each layer in the decoder consists of two operations: deconvolution and concatenation. The deconvolution process upsamples the feature maps from the encoder to obtain higher resolution image features. In the concatenation operation, the upsampled feature maps obtained through deconvolution are combined with the corresponding layer's feature maps from the encoder. By combining the deconvolution and concatenation operations in the decoder, the network can obtain more detailed and contextually rich feature maps. This allows the decoder to generate more accurate and visually appealing image results.

During the deconvolution process, the lack of contextual information may lead to the loss of some fine details in RGB images, thereby affecting the results of image depth estimation. To address this issue, this paper incorporates a Non-local operation attention mechanism, which calculates the similarity of each pixel to weight the context information of each pixel. By doing so, the network can capture and utilize richer contextual information during the deconvolution process, mitigating the loss of fine details and enhancing the accuracy of image depth estimation.

The specific structure of the decoder network model for the end-to-end network is shown in Table 2. The experimental results show that incorporating a Non-local operation attention mechanism between [*upconv*6, *conv*5*b*] yields the best performance. In image depth estimation, to obtain four different scales of depth maps and upsample the first three scales, bilinear interpolation is commonly used. The sampling rates for the first three scales are 1/4, 1/2, and 1, respectively, when performing bilinear interpolation. Finally, by fusing the three scales of depth maps, the network obtains the final set of four different scales of depth maps [*disp*1, *disp*2, *disp*3, *disp*4].

**Table 2:** Decoder network model specific structure.

**Name**	**Input**	**Kernel size**	**Stride**
upconv7	conv7b	3 × 3	2
iconv7	[upconv7, conv6b]	3 × 3	1
upconv6	iconv7	3 × 3	2
context	Non-Local Block [upconv6, conv5b]		
iconv6	context	3 × 3	1
upconv5	iconv6	3 × 3	2
iconv5	[upconv5, conv4b]	3 × 3	1
upconv4	iconv5	3 × 3	2
iconv4	[upconv4, conv3b]	3 × 3	1
disp4	iconv4, sigmoid	3 × 3	1
disp4_up	disp4, bilinear	H/4, W/4	
upconv3	iconv4	3 × 3	2
iconv3	[upconv3, conv2b, disp4_up]	3 × 3	1
disp3	iconv3, sigmoid	3 × 3	1
disp3_up	disp3, bilinear	H/2, W/2	
upconv2	iconv3	3 × 3	2
iconv2	[upconv2, conv1b, disp3_up]	3 × 3	1
disp2	iconv2, sigmoid	3 × 3	1
disp2_up	disp2, bilinear	H, W	
upconv1	iconv2, sigmoid	3 × 3	2
iconv1	[upconv1, disp2_up]	3 × 3	1
disp1	iconv1, sigmoid	3 × 3	1
output	[disp1, disp2, disp3, disp4]		

### 2.3. Non-local attention mechanism

In computer vision, incorporating attention mechanisms can help models focus on more important areas of an image, thereby reducing the influence of irrelevant background. Non-local operation is a type of attention mechanism that uses global information to capture long-range dependencies between pixels in an image. Compared to local operations, Non-local operations have a broader receptive field and stronger modeling capabilities. The fundamental concept behind non-local operations is to compute the similarity between each pixel and all other pixels in the image. These similarities are then used to adaptively weight the entire image, allowing the model to better understand the global structure of the image.

**Figure 7:**
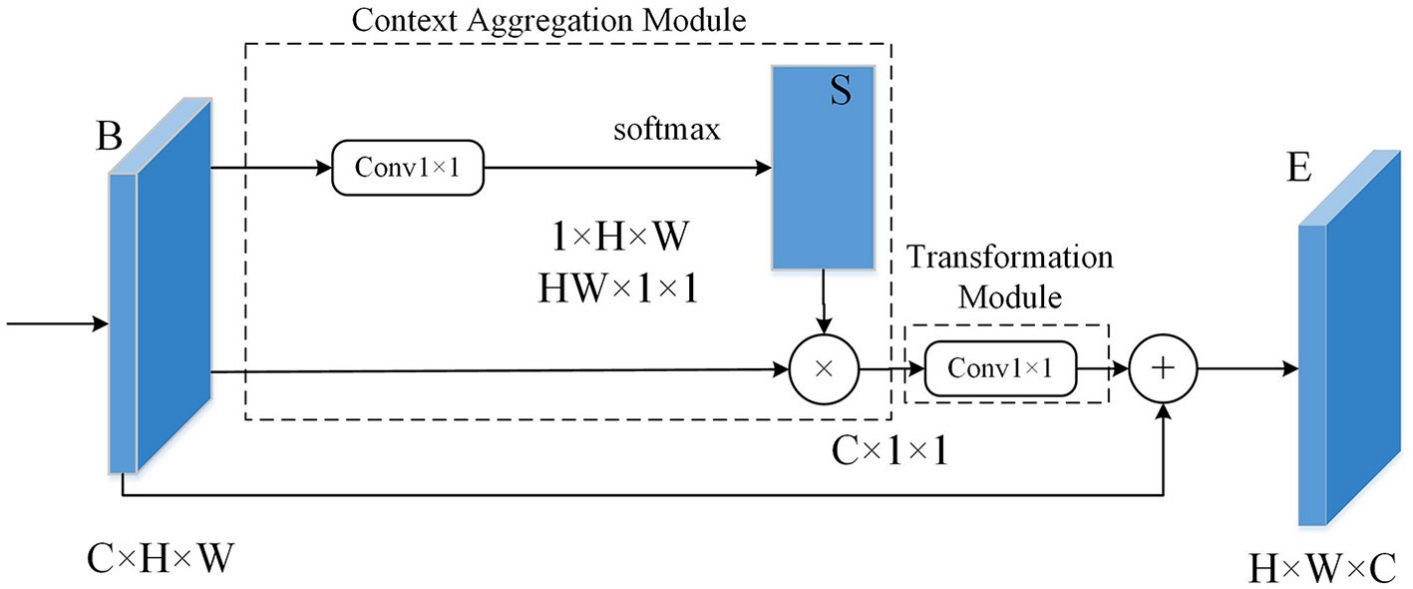
Schematic diagram of the non-local operation module.

Figure 7 shows the schematic diagram of the non-local operation module. In this paper, both the Context Aggregation Module and the Transformation Module have incorporated 1 × 1 convolutions, which can reduce the dimensionality of the input feature map without losing information. The Context Aggregation Module is the core component for implementing non-local operations. Its main function is to measure the relationship between two pixels by calculating metrics such as Euclidean distance or cosine similarity. By computing these metrics, the Context Aggregation Module can determine the similarity or dissimilarity between pixels in the input feature map. This allows the module to capture long-range dependencies and establish the global context within the image, enabling the model to understand the relationships between different pixels and extract important contextual information. The Transformation Module is used to convert the input feature map into a new feature map for further processing. The output of the Transformation Module serves as the input to the next layer, enabling communication and integration of data across different layers. 1 × 1 convolutions have two main purposes: first, to reduce dimensions and decrease the number of channels; second, to introduce non-linear elements to enhance the expressive capability of neural networks.

The mathematical definition of the non-local operation is as follows:


(4) $$\begin{array}{l} y_{i}=\frac{1}{C\left(x\right)}\underset{\forall j}{\sum }f\left(x_{i},x_{j}\right)g\left(x_{j}\right) \end{array}$$

In the above Equation: (1) *x* is feature map; (2) *i* represents a spatial position of a point on the input *x* or output *y*; (3) The response value at position *i* is represented by *y*_*i*_; (4) The variable *j* iterates over the spatial coordinates of all points on the input x or output *y*; (5) The variable *x*_*j*_ represents the value at position *j* on the input data; (6) The function *f*(*x*_*i*_, *y*_*j*_) calculates the similarity between position *i* and position *j* of the input data; (7) The function *g*(*x*_*j*_) calculates a representation of the input data at position *j*, which can be understood as a weight for the similarity function *f*(*x*_*i*_, *y*_*j*_); and (8) The final response value *y* of the Non-local operation at position *i* is obtained by summing the weighted similarities *f*(*x*_*i*_, *y*_*j*_)*g*(*x*_*j*_) of each position *j* relative to the current position *i*. This sum is then normalized using the normalization factor *C*(*x*), which results in the weighted sum of features from all positions being used as the response value at that specific position.

### 2.4. Deep smoothing loss function

To reduce errors and uncertainties of the results, smooth constraints can be used in depth estimation. Smooth constraints refer to the reduction of noise and discontinuities in the depth map by limiting the differences between the depths of neighboring pixels. This can be accomplished by adding a smoothing term to the loss function of the depth estimation model. To further improve the accuracy and effect of depth estimation, this paper improves the loss function of the model and adopts a depth smoothing loss. The smoothness error of this loss function can be obtained by calculating the gradients of the depth map. To better represent the variations in depth, the gradient computation is performed in the logarithmic domain of the depth map. Based on experience, discontinuous depth values in the depth map are typically found at the edges of the image. Therefore, the edge of the image to be estimated is used as a penalty factor to limit the smoothing loss. The deep smoothing loss constructed in this paper includes the following three aspects:

(1) Smoothing loss based on gradient computation of the depth map. By computing the gradient of the depth map in the logarithmic domain, we can obtain information about depth variations, thereby enhancing the smoothness of the depth map.


(5) $$\begin{array}{l} \partial_{x}Z_{\log}^{i,j}=Z_{\log}^{i,j}-Z_{\log}^{i+1,j},i=0,1\cdots W-1;j=0,1\cdots H-1 \end{array}$$

(6) $$\begin{array}{l} \partial_{x}Z_{\log}^{i,j}=Z_{\log}^{i,j}-Z_{\log}^{i,j+1},i=0,1\cdots W-1;j=0,1\cdots H-1 \end{array}$$

(7) $$\begin{array}{l} \nabla Z_{\log}=|\partial_{x}Z_{\log}|+|\partial_{y}Z_{\log}| \end{array}$$

In the above equation, ∇*Z*_*log*_ represents the logarithmic gradient of the depth map, ∂_*x*_*Z*_*log*_ denotes the gradient's horizontal component, and ∂_*y*_*Z*_*log*_ corresponds to the gradient's vertical component. The indices *i* and *j* represent the row and column indices of the depth map, respectively, while *W* and *H* represent the width and height of the depth map.

(2) Smoothing Loss based on Edge information. By utilizing the edge information from the input image as a constraint, the depth map can undergo a more accurate smoothing process.


(8) $$\begin{array}{l} \nabla I_{gray}=|\partial_{x}I_{gray}|+|\partial_{y}I_{gray}| \end{array}$$

In the equation, *I*_*gray*_ represents the grayscale image obtained from the RGB image, where each pixel value lies in the range of 0 to 255. ∂_*x*_*I*_*gray*_ denotes the horizontal gradient, and ∂_*y*_*I*_*gray*_ represents the vertical gradient.

(3) Final depth map smoothing loss. As shown in Equation (9):


(9) $$\begin{array}{l} L_{smoth}=\frac{1}{N}\underset{i,j}{\sum }\left(\nabla Z_{\log}^{i,j}\cdot e^{-\nabla I_{gray}^{i,j}}\right) \end{array}$$

In the equation, *N* represents the total number of pixels in the image.

## 3. Experiment

### 3.1. Experimental environment and process

In this study, the improved unsupervised depth estimation network model was implemented and trained using the PyTorch deep learning framework on an NVIDIA GTX3090 GPU. The experiments were conducted to evaluate the model's performance in depth estimation. In addition to using the publicly available KITTI dataset (Geiger et al., 2013), this study also utilized a dataset collected from a catadioptric panoramic camera for the experiments. During the experimental process, batch normalization layers and the Adam optimizer were applied to all layers except the input layer. In the Adam optimizer, set β_1_ = 0.95, β_2_ = 0.994, the learning rate to 0.001, and the mini-batch size to 3. Batch normalization layers are applied to every layer except for the input layer, which helps accelerate the training of the network and improve its accuracy. Moreover, a relatively small mini-batch size was chosen to facilitate faster convergence of the network. Table 3 shows the parameters of the experimental environment in this chapter.

**Table 3:** Experimental environment parameters.

**Project**	**Environment configuration**	**Version**	**Quantity**
Operating system	Windows10	21H2	-
Deep learning framework	PyTorch	1.12.0	-
GPU	Nvidia	GTX3090	1
Programming languages	Python	3.10	-
Public datasets	Kitti	-	10,000
Self-built dataset	-	-	1,000

### 3.2. Evaluation index

This paper employs four evaluation metrics to assess the model's performance, namely Absolute Relative Error (AbsRel), Squared Relative Error (SqRel), Root Mean Squared Error (RMS), and Log Error (Log). The specific form is as follows:

AbsRel: The absolute relative error is a metric used to evaluate the difference between the model's predicted values and the ground truth values. Its calculation formula is the absolute difference between the predicted value and the ground truth value, divided by the ground truth value, reflecting the magnitude of the error relative to the ground truth value.


(10) $$\begin{array}{l} Abs\text{Re}l=\frac{1}{N}\overset{N}{\underset{i=1}{\sum }}\frac{\left|D_{i}-D_{i}^{*}\right|}{D_{i}^{*}} \end{array}$$

SqRel: The squared relative error is computed by taking the square of the difference between the predicted value and the ground truth value, and then dividing it by the ground truth value.


(11) $$\begin{array}{l} Sq\text{Re}l=\frac{1}{N}\frac{{\left|D_{i}-D_{i}^{*}\right|}^{2}}{D_{i}^{*}} \end{array}$$

RMS: The root mean square error is a metric that calculates the square root of the mean of the squared prediction errors. It measures the average magnitude of the prediction errors and is commonly used to evaluate the accuracy of a model's predictions.


(12) $$\begin{array}{l} RMS=\sqrt{\frac{1}{N}\overset{N}{\underset{i=1}{\sum }}|D_{i}-D_{i}^{*}{|}^{2}} \end{array}$$

Log: The logarithmic error is a metric that first takes the logarithm of the predicted values and the true values and then calculates the error between these logarithms. This metric is useful when dealing with data that has a large range or significant differences between values.


(13) $$\begin{array}{l} Log=\frac{1}{N}\overset{N}{\underset{i=1}{\sum }}|\mathrm{lg}D_{i}-\mathrm{lg}D_{i}^{*}| \end{array}$$

In the above expressions, *N* represents the total number of valid pixels used for evaluation across all RGB images. *D*_*i*_ denotes the predicted depth of the *i*-th pixel in the RGB image, and $D_{i}^{*}$ represents the true depth of the same pixel.

### 3.3. Results and analysis

The improved concentric circle approximate expansion algorithm is used to process the panorama and extract the effective area. Creating a dataset from these image segments and performing depth estimation, which validates the robustness of the improved algorithm proposed in this paper.

**Figure 8:**
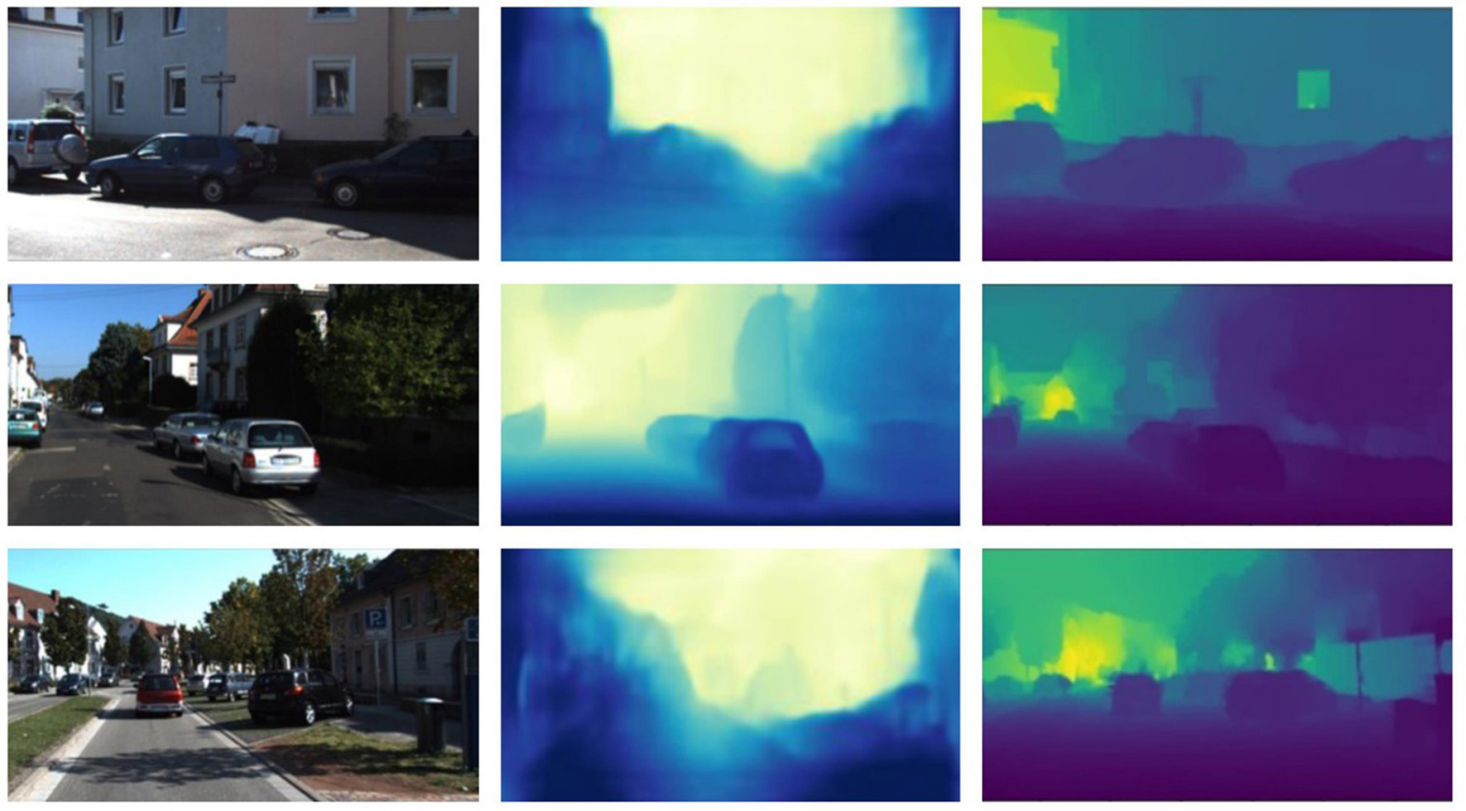
Results of depth estimation.

Figure 8 shows the result of depth estimation. In Figure 8, the first column is the original image, the second column represents the depth estimation results by Zhou et al. (2017), and the last column shows the depth map obtained using the method proposed in this paper. The darker colors in the depth map indicate closer distances, while lighter colors represent farther distances. Through experiments on different scene images, the depth estimation results of the original algorithm are fuzzy, and cannot get accurate results in most cases. The method improved in this paper can generate clearer depth maps. Especially in the case of edge segmentation of objects, the effect of the proposed method is more obvious.

To validate the effectiveness of the improved depth estimation algorithm proposed in this paper, experiments and analyses were conducted on the Kitti dataset. The proposed depth estimation model was evaluated by comparing it with the depth estimation models introduced by Eigen et al. (2014) and Zhou et al. (2017). The experimental results comparison is shown in Table 4.

**Table 4:** Comparison with other methods.

**Methods**	**AbsRel**	**SqRel**	**RMS**	**Log**	**Dataset**
Eigen	0.204	1.385	5.995	0.283	Kitti
Zhou	0.202	1.347	5.679	0.264	Kitti
In this paper	0.196	1.423	6.237	0.269	Kitti

As shown in Table 4, our proposed method exhibits lower absolute relative error and log error compared to the supervised approach by Eigen et al. (2014), with reductions of 0.8 and 1.4%, respectively. Compared to the unsupervised learning method by Zhou et al. (2017), our approach performs better in terms of absolute relative error, with a reduction of 0.6%, but exhibits slightly higher overall error. In conclusion, our improved method in this paper exhibits better performance in terms of error, with higher accuracy and the ability to address the blurriness issue in image depth estimation.

**Figure 9:**
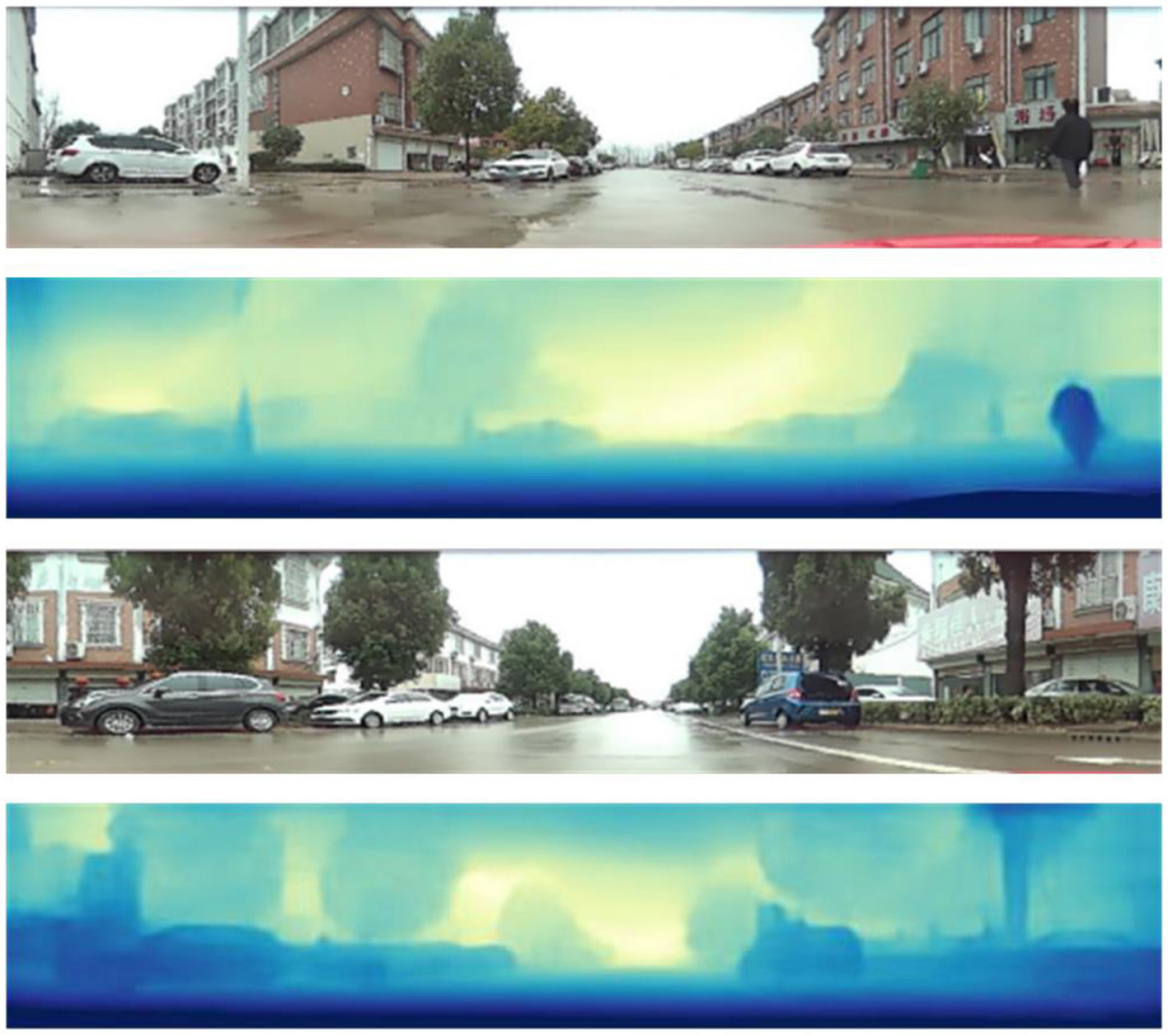
Highway depth estimation results.

**Figure 10:**
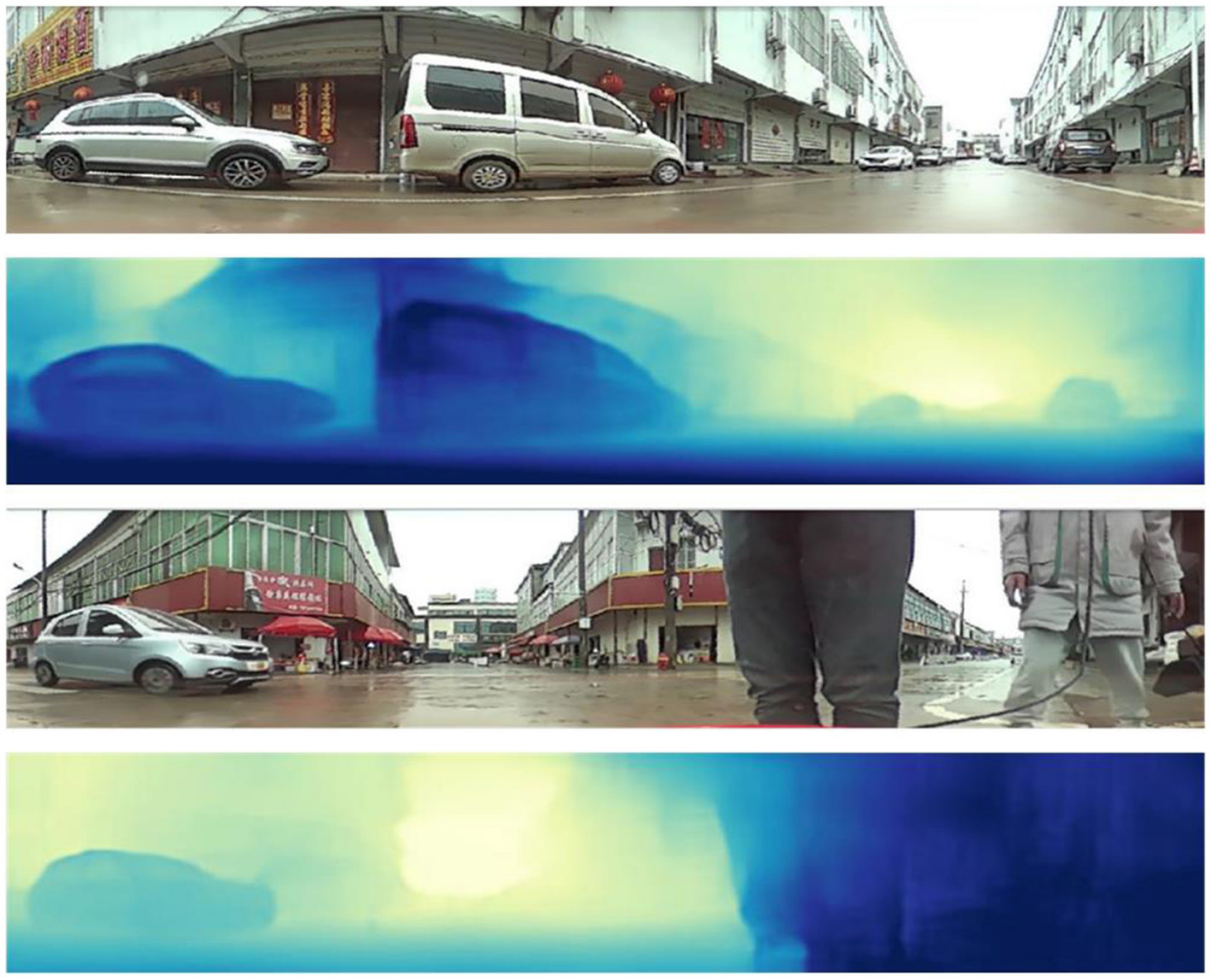
Neighborhood street depth estimation results.

To further validate the effectiveness of our algorithm, we conducted tests on 200 images captured by the catadioptric panoramic camera in various scenes. Figures 9, 10 show some of the experimental results from different scenes.

In Figures 9, 10, the first and third rows show the original test images. The second and fourth rows display the depth estimation results. The color of the pixels in the depth estimation images represents the distance, where darker colors indicate closer distances and lighter colors indicate farther distances.

From the experimental results, it can be observed that the improved image depth estimation algorithm in this paper can relatively accurately estimate the depth range of objects in the images. Considering the distance analysis relative to the vehicle during image capture, for objects such as vehicles and pedestrians located within a distance of less than 2.5 meters, their corresponding depth values in the depth map fall within the range of 0 to 80, which shows the darkest colors in the depth map; for objects with a distance of 2.5 to 4 meters, the gray values in the depth map results fall within the range of 81 to 150; for objects with a distance greater than 4 meters, the gray values in the depth map results fall within the range of 151 to 255, which results in relatively lighter colors in the depth map.

In conclusion, the research approach proposed in this paper, based on the catadioptric panoramic camera, has demonstrated its effectiveness in depth estimation.

## 4. Conclusion

This paper proposes a monocular depth estimation algorithm based on the catadioptric panoramic camera. The paper proposes an improved concentric circle approximation unwrapping algorithm to process the panoramic images captured by the catadioptric panoramic camera. This algorithm is used to unwrap the distorted panoramic images into a more usable format for further analysis and depth estimation. The proposed approach enhances the quality and accuracy of the panoramic data. The effective region is extracted according to the unfolded rectangular panorama characteristics. Finally, this paper proposes a new unsupervised end-to-end depth estimation network model. The experimental results show that the depth estimation results of the proposed algorithm are better than the existing algorithms.

## Data availability statement

The raw data supporting the conclusions of this article will be made available by the authors, without undue reservation.

## Author contributions

FY: Writing—original draft, Writing—review and editing. LL: Writing—original draft, Writing—review and editing. XD: Writing—original draft. QZ: Writing—review and editing. YL: Writing—review and editing, Writing—original draft.
